# Reduced ex vivo release of pro-inflammatory cytokines and elevated plasma interleukin-6 are inflammatory signatures of post-stroke delirium

**DOI:** 10.1186/s12974-018-1156-y

**Published:** 2018-04-18

**Authors:** Katarzyna Kowalska, Elzbieta Klimiec, Kazimierz Weglarczyk, Joanna Pera, Agnieszka Slowik, Maciej Siedlar, Tomasz Dziedzic

**Affiliations:** 1 Department of Neurology, Jagiellonian University Medical College, ul. Botaniczna 3, 31-503 Krakow, Poland; 2 Department of Clinical Immunology, Institute of Paediatrics, Jagiellonian University Medical College, Krakow, Poland

**Keywords:** Delirium, Cytokines, Stroke, Inflammation

## Abstract

**Background:**

Experimental studies suggest that systemic inflammation contributes to the pathophysiology of delirium. The aim of our study was to determine blood-derived inflammatory signatures of post-stroke delirium.

**Methods:**

We included 144 ischemic stroke patients. We assessed delirium on a daily basis during the first 7 days of hospitalization. Venous blood was collected at day 3 after the onset of stroke and stimulated ex vivo with lipopolysaccharide (LPS). We measured LPS-induced cytokine concentration (TNFα, IP-10, IL-1β, IL-6, IL-8, IL-10, and IL-12p70) as well as plasma levels of IL-6 and TNFα.

**Results:**

Delirium was diagnosed in 21.5% of patients. After correction for monocyte count, patients with delirium had reduced LPS-induced TNFα, IP-10, IL-1β, IL-6, and IL-12 release. The plasma IL-6 level was higher in delirious patients compared to patients without delirium. After adjusting for stroke severity and infections, higher ex vivo TNFα (OR 0.29, 95%CI 0.11–0.72, *P* = 0.01), IP-10 (OR 0.25, 95%CI 0.08–0.73, *P* = 0.01), IL-1β (OR 0.42, 95%CI 0.20–0.89, *P* = 0.02), and IL-12 (OR 0.07, 95%CI 0.01–0.70, *P* = 0.02) release was associated with the reduced risk of delirium. In multivariate analysis, the higher plasma IL-6 was associated with the increased risk of delirium (OR 1.61, 95%CI 1.00–2.58, *P* = 0.04).

**Conclusions:**

Reduced ex vivo release of pro-inflammatory cytokines after LPS stimulation and the elevated plasma IL-6 are signatures of post-stroke delirium.

## Background

Delirium is a neuropsychiatric syndrome characterized by acute, fluctuating changes in attention, awareness, and cognition. Delirium is common among hospitalized elderly people and is associated with increased mortality and poor functional and cognitive outcome [1]. An estimated rate of delirium after stroke is 26% with a range of 10 to 48% [2].

The pathophysiology of delirium is still poorly understood. One of the current hypotheses of delirium suggests that acute peripheral inflammation induces activation of brain parenchymal cells and an expression of pro-inflammatory mediators in the brain. These neuroinflammatory changes evoke a synaptic and neuronal dysfunction and subsequent behavioral and cognitive symptoms [3–5]. The elderly and patients with neurodegenerative diseases are particularly prone to develop delirium triggered by systemic inflammation [3].

Although systemic inflammation is recognized as a trigger of delirium, there is still limited evidence that links circulating inflammatory mediators with delirium in humans. The most replicated finding, albeit not confirmed by some researchers, is the elevated blood interleukin (IL)-6 level during delirium [6–9]. Other blood-based inflammatory mediators which predicted delirium or were elevated during delirium in some studies include C-reactive protein (CRP) [10, 11], IL-1β [12], IL-2 [9, 13], IL-8 [7], tumor necrosis factor alpha (TNFα) [9, 13], and soluble TNFα receptor 1 and 2 [12, 14].

Identification of blood-derived inflammatory signatures of delirium is important for better understanding interactions between the immune system and delirium and searching of potential biomarkers of delirium. In this study, we aimed to determine inflammatory signatures of post-stroke delirium. Toll-like receptor 4 (TLR4) is a master regulator of innate immunity. We assessed ex vivo cytokine release after whole blood stimulation with lipopolysaccharide (LPS), an agonist of TLR4. For comparison, we measured plasma levels of two cytokines: IL-6 and TNFα. We choose IL-6 because numerous studies demonstrated that the blood level of this cytokine is elevated in acute stroke patients and correlates with poor outcome [15]. Tumor necrosis factor alpha was chosen, because a few studies showed an associated between this cytokine and post-stroke infections [16].

## Methods

### Patient selection and clinical assessment

The participants for this study were prospectively recruited from stroke patients hospitalized in the Department of Neurology, University Hospital, Krakow, Poland, between October 2016 and June 2017. The inclusion criteria were as follows: (1) ischemic stroke, (2) time from the onset of stroke symptoms onset to admission < 24 h, (3) pre-stroke modified Rankin Scale score 0–2 (independence in daily activities), (4) National Institute of Health Stroke Scale (NIHSS) score on admission > 3, and (5) informed patient consent. The exclusion criteria were as follows: (1) chronic inflammatory, autoimmune or cancerous diseases; (2) coma; and (3) severe aphasia disabling the assessment of delirium’s core features. Written informed consent was obtained from each patient included in the study. The study protocol conforms to the ethical guidelines of the 1975 Declaration of Helsinki. The study protocol was approved by the Bioethics Committee of Jagiellonian University.

Since the main goal of our study was to determine inflammatory signatures that are present *during* an episode of delirium, we excluded patients in whom delirium occurred before or after day 3 (i.e. the day of blood sampling), but who had no delirium’s episode at day 3.

The diagnosis of pneumonia required the presence of clinical findings (at least two from the following: productive cough and/or purulent sputum, rales, fever, and leukocytosis), as well as pulmonary infiltrates in chest X-ray. Symptomatic urinary tract infection was diagnosed based on one of the following two sets of criteria: (1) at least one of the following signs is present: fever (> 38 °C), urgency, dysuria, or suprapubic tenderness *and* positive urine culture (≥ 10^5^ microorganisms per cubic centimetre with no more than two species of microorganisms), or (2) at least one of the following signs is present: fever (> 38 °C), urgency, dysuria, or suprapubic tenderness *and* at least two of the following: positive dipstick for leukocyte esterase and/or nitrate, pyuria (urine specimen with ≥ 10 white blood cell/mm^3^), and organisms seen in Gram stains of unspun urine [17].

Patients were screened for delirium on daily basis during first 7 days after admission. Core features of delirium were assessed using Brief Confusion Assessment Method (bCAM) for verbal patients [18] and Intensive Care Units version (CAM-ICU) for non-verbal patients [19]. The final diagnosis of delirium was based on DMS-5 criteria [20].

The Informant Questionnaire on Cognitive Decline in the Elderly (IQCODE) was used to diagnose a pre-stroke cognitive decline [21]. IQCODE consists of 26 items that rate the change in patients’ intellectual abilities over the past 10 years. A cutoff of 3.3 was chosen to diagnose a pre-stroke cognitive decline [22].

No formal protocol was used to treat delirium. In general, management of delirium included early mobilization, treatment of infections and pain, control of fluids and electrolytes, and use of visual and hearing aids in patients with sensory impairment. Neuroleptics were given only to patients with severe agitation.

### Laboratory assays

To avoid diurnal variation in cytokine production, blood was obtained at day 3 after stroke rather than upon admission. Venous blood was collected in heparinized tubes (Sarstedt, Germany) between 7:00 AM and 7:30 AM. The whole blood was diluted by 1:5 in sterile RPMI 1640 medium supplemented with l-glutamine (Sigma Aldrich, St. Louis, MO) and stimulated for 4 h at 37 °C and 5%CO_2_ with LPS (10 ng/mL, *E. coli* 0111:B4, Sigma Aldrich, St. Louis, MO) in sterile tubes (Lonza, Walkersville, MD). The supernatants were removed and stored at − 80 °C until further analysis.

Similar to some previous studies [17, 23, 24], LPS-induced cytokines were normalized for monocyte count, because monocytes are the main producers of cytokines after whole blood stimulation with endotoxin [25]. Moreover, normalization by monocyte count reduced the coefficient of variation [26]. Taking into account the previous publications [17, 23, 27] and our own preliminary experiments on kinetics of cytokine release after whole blood stimulation, a stimulation was performed for 4 h for TNFα and interferon-gamma-inducible protein 10 (IP-10) and 24 h for interleukin(IL)-1β, IL-6, IL-8, IL-10, and IL-12p70. TNFα and IP-10 concentration was measured using commercially available ELISA kits from R&D Systems (Minneapolis, MN). Concentration of IL-1β, IL-6, IL-8, IL-10, and IL-12p70 was determined by a cytometric bead array immunoassay (Human Inflammatory Kit, BD Bioscience, San Diego, CA).

Plasma level of TNFα and IL-6 was measured using ELISA kits from R&D Systems (Minneapolis, MN). Cytokine detection limits were 0.19 pg/mL for TNFα and 0.11 pg/mL for IL-6.

### Statistical analysis

The *χ*^2^ test was used to compare proportions, while the Mann–Whitney *U* test was used to compare continuous variables between groups. Logistic regression was used to determine the predictors of functional outcome. Distribution of cytokines was standardized to a mean 0 and standard deviation of 1. NIHSS score on admission were put to a model as continuous variables. Variables with *P* < 0.05 in univariate analysis were included in multivariate analysis. The Box-Tidwell test was used to check the linearity of the logit for the continuous independent variables in logistic regression analysis. The calculations were performed using the program STATISTICA for Windows (version 12.5, Statsoft, Poland). The calculated odds ratios are per standard deviation.

In additional analysis, we assessed an association between raw values of LPS-induced cytokine (not normalized for monocyte count) and delirium. We used the receiver operating characteristic curves to find an optimal cutoff level of cytokines that differentiates delirious patients from non-delirious patients.

## Results

We recruited 157 stroke patients. From final analysis, we excluded patients who had no delirium at day 3 (i.e. the day of blood collection), but who developed delirium before (*N* = 10) or after day 3 (*N* = 3).

The final cohort included 144 patients (median age 69, IQs 63–79; 57.6% male). Delirium was diagnosed in 21.5% of patients. Baseline characteristics of the patients with delirium and the patients without delirium are shown in Table 1.

**Table 1:** Characteristics of patients with delirium and patients without delirium

	Patients with delirium (*N* = 31)	Patients without delirium (*N* = 113)	*P* value
Age, median (IQs)	71 (64–80)	68 (63–79)	0.57
Male, *n* (%)	18 (58.1)	65 (57.5)	0.96
Hypertension, *n* (%)	24 (77.4)	82 (72.6)	0.59
Diabetes mellitus, *n* (%)	8 (25.8)	30 (26.5)	0.93
Atrial fibrillation, *n* (%)	13 (41.9)	32 (28.3)	0.15
Myocardial infarction, *n* (%)	5 (16.1)	14 (12.4)	0.58
Previous stroke, *n* (%)	3 (9.7)	14 (12.4)	0.68
IQCODE > 3.3, *n* (%)^a^	3/24 (12.5)	9/85 (10.6)	0.79
NIHSS score on admission, median (IQs)	17 (15–20)	7 (5–15)	< 0.01
Pneumonia, *n* (%)	9 (29.0)	3 (2.6)	< 0.01
Urinary tract infection, *n* (%)^b^	13/30 (43.3)	20/99 (20.2)	0.01
White blood cells count, × 10^3^/μL, median (IQs)	11.7 (9.3–12.9)	8.3 (6.6–9.6)	< 0.01
Monocyte count, × 10^3^/μL, median (IQs)	1.1 (0.9–1.2)	0.8 (0.6–1.0)	< 0.01
Intravenous thrombolysis, *n* (%)	20 (64.5)	64 (56.6)	0.43
Mechanical thrombectomy, *n* (%)	7 (22.6)	24 (21.2)	0.87

*IQs* interquartiles, *IQCODE* the Informant Questionnaire on Cognitive Decline in the Elderly

^a^Data available for 109 patients

^b^Data available for 129 patients

Patients with delirium had more severe neurological deficit on admission and more often suffered from pneumonia and urinary tract infection.

Compared to patients without delirium, delirious patients exhibited reduced release of TNFα, IP-10, IL-1β, IL-6, and IL-12 after whole blood stimulation with LPS (Table 2). The plasma level of IL-6, but not TNFα, was higher in patients with delirium.

**Table 2:** Cytokine levels in patients with delirium and patients without delirium

	Patients with delirium (*N* = 31)	Patients without delirium (*N* = 113)	*P* value
Ex vivo stimulation			
TNFα, pg/10^3^ monocytes	1.76 (1.47–2.32)	3.17 (2.31–4.42)	< 0.01
IP-10, pg/10^3^ monocytes	0.17 (0.10–0.46)	0.56 (0.32–0.92)	< 0.01
IL-1β, pg/10^3^ monocytes	1.12 (0.62–1.78)	1.85 (1.26–2.61)	< 0.01
IL-6, pg/10^3^ monocytes	10.37 (8.55–14.52)	14.21 (9.81–18.16)	0.02
IL-12, pg/10^6^ monocytes	0 (0–0)	1.46 (0–8.57)	< 0.01
IL-10, pg/10^6^ monocytes	66.58 (39.57–86.54)	59.70 (36.85–99.02)	0.82
IL-8, pg/10^3^ monocytes	2.03 (1.51–2.93)	1.64 (1.08–2.56)	0.13
TNFα, pg/mL	2066 (1578–2550)	2404 (1555–3869)	0.03
IP-10, pg/mL	166.97 (108.45–366.40)	436.10 (201.28–797.10)	< 0.01
IL-1β, pg/mL	1153 (680–1690)	1325 (1046–2092)	0.11
IL-6, pg/mL	10,455 (8885–16,362)	10,407 (7280–13,319)	0.41
IL-12, pg/mL	0 (0–0)	1.48 (0–6.91)	< 0.01
IL-10, pg/mL	69.23 (36.67–103.38)	46.62 (31.99–72.11)	0.06
IL-8, pg/mL	2240 (1469–3209)	1279 (872–2055)	< 0.01
Plasma			
TNFα, pg/ml	0.91 (0.53–1.27)	0.70 (0.36–1.12)	0.09
IL-6, pg/mL	17.95 (7.09–46.75)	3.94 (2.21–7.29)	< 0.01

Data are shown as medians with interquartiles

The results of univariate analysis and multivariate analysis are shown in Table 3.

**Table 3:** Results of uni- and multivariate analysis

	Univariate analysis	Multivariate analysis^a^
Ex vivo stimulation		
TNFα	OR 0.19, 95%CI 0.09–0.41, P < 0.01	OR 0.29, 95%CI 0.11–0.72, P = 0.01
IP-10	OR 0.13, 95%CI 0.04–0.38, P < 0.01	OR 0.25, 95%CI 0.08–0.73, P = 0.01
IL-1β	OR 0.34, 95%CI 0.18–0.67, P < 0.01	OR 0.42, 95%CI 0.20–0.89, P = 0.02
IL-6	OR 0.62, 95%CI 0.40–0.96, P = 0.03	OR 0.76, 95%CI 0.43–1.35, P = 0.35
IL-12	OR 0.06, 95%CI 0.01–0.48, P = 0.01	OR 0.07, 95%CI 0.01–0.70, P = 0.02
IL-10	OR 0.85, 95%CI 0.55–1.30, P = 0.44	OR 1.00, 95%CI 0.59–1.69, P = 0.99
IL-8	OR 1.15, 95%CI 0.80–1.66, P = 0.43	OR 1.34, 95%CI 0.80–2.24, P = 0.26
Plasma		
TNFα	OR 1.16, 95%CI 0.81–1.68, P = 0.41	OR 0.89, 95%CI 0.53–1.48, P = 0.64
IL-6	OR 2.77, 95%CI 1.53–5.04, P < 0.01	OR 1.61, 95%CI 1.00–2.58, P = 0.04

^a^Adjusted for NIHSS score on admission, pneumonia, and urinary tract infection

ORs are per standard deviation

In univariate analysis, higher release of TNFα (OR 0.19, 95%CI 0.09–0.41, *P* < 0.01), IP-10 (OR 0.13, 95%CI 0.04–0.38, *P* < 0.01), IL-1β (OR 0.34, 95%CI 0.18–0.67, *P* < 0.01), IL-6 (OR 0.62, 95%CI 0.40–0.96, *P* = 0.03), and IL-12 (OR 0.06, 95%CI 0.01–0.48, *P* = 0.01) was associated with the reduced risk of delirium. Higher plasma IL-6 level was associated with the increased risk of delirium (OR 2.77, 95%CI 1.53–5.04, *P* < 0.01).

An association between delirium and reduced release of TNFα (OR 0.29, 95%CI 0.11–0.72, *P* = 0.01), IP-10 (OR 0.25, 95%CI 0.08–0.73, *P* = 0.01), IL-1β (OR 0.42, 95%CI 0.20–0.89, *P* = 0.02), and IL-12 (OR 0.07, 95%CI 0.01–0.70, *P* = 0.02) as well as the increased plasma IL-6 (OR 1.61, 95%CI 1.00–2.58, *P* = 0.04) remained significant after adjusting for stroke severity (NIHSS score on admission), pneumonia, and urinary tract infection.

Finally, we analysed an association between delirium and raw values of LPS-induced cytokine (not normalized for monocyte count). Delirious patients had reduced release of TNFα, IP-10, and IL-12 and increased release of IL-8 compared to patients without delirium (Table 2). In univariate analysis, TNFα < 2857.6 pg/mL (OR 7.86, 95%CI 2.27–27.22, *P* < 0.01), IP-10 < 333.4 pg/mL (OR 4.30, 95%CI 1.99–9.28, *P* < 0.01), IL-12 < 0.30 pg/mL (OR 2.78, 95%CI 1.35–5.72, *P* < 0.01), IL-10 > 57.0 pg/mL (OR 2.52, 95%CI 1.23–5.16, *P* = 0.01), and IL-8 > 1786.4 pg/mL (OR 5.00, 95%CI 2.36–10.60, *P* < 0.01) were associated with delirium. An association between delirium and TNFα < 2857.6 pg/mL (OR 6.14, 95%CI 1.52–24.73 *P* = 0.01), IP-10 < 333.4 pg/mL (OR 2.61, 95%CI 1.04–6.55, *P* = 0.04), IL-10 > 57.0 pg/mL (OR 3.72, 95%CI 1.46–9.47, *P* = 0.01), and IL-8 > 1786.4 pg/mL (OR 5.18, 95%CI 2.08–12.93, *P* < 0.01) remained significant after adjusting for NIHSS score, pneumonia, and urinary tract infection.

## Discussion

We found reduced release of TNFα, IP-10, IL-1β, and IL-12 after ex vivo blood stimulation and the elevated plasma IL-6 level in delirious patients.

To our best knowledge, it is the first report showing diminished ex vivo release of pro-inflammatory cytokines in patients with delirium. This finding is consistent with transient post-stroke immunodepression [28]. In acute stroke patients, circulating monocytes exhibit an endotoxin tolerance status that manifests as the reduced TNFα synthesis and the diminished HLA-DR expression after LPS challenge [29]. An endotoxin tolerant state is a phenomenon observed in patients with different pathologies including sepsis, acute coronary syndrome, heart failure, and cancer [30]. Endotoxin tolerance is a key mechanism to limit an excessive innate reaction to infection or injury. It was suggested that post-stroke immunodepression is triggered by over-activation of the sympathetic nervous system and the release of damage-associated molecular patterns (DAMPs) [29, 31]. It would be interesting to examine in future studies a relationship between markers of autonomic nervous systems, circulating DAMPs and delirium.

Other mechanism which could be responsible for post-stroke immunodepression is a release of high mobility group box 1 (HMGB1) from the necrotic brain tissue to blood. In vitro, extracellular HMGB1 induced endotoxin tolerance in macrophages [32]. In the animal model of cerebral ischemia, circulating HMGB1 acting via the receptor of advanced glycation end-products (RAGE) was responsible for the reduced secretion of TNFα and IL-12 by monocytes and lymphopenia [33]. A relationship between delirium and circulating HMGB1 requires further investigation.

We confirmed and extended previous studies examining an association between IL-6 and delirium. IL-6 is the strongest activator of the hepatic acute phase reaction, and its blood levels are elevated in many infectious and inflammatory diseases [34]. Higher blood levels of IL-6 were observed among delirious patients after cardiac surgery [8], hip fracture [7], and major elective surgery [9] as well as older patients acutely admitted to the hospital [6]. In stroke patients, a source of circulating IL-6 seems to be an ischemic brain or an adipose tissue [35].

In our study, plasma TNFα level did not differ significantly between patients with delirium and patients without delirium. The studies examining an association between this cytokine and delirium yielded conflicting results. A few studies reported the elevated level of circulating TNFα in patients with post-operative delirium [9, 13] whereas other studies did not find such as an association [7, 36].

The additional analysis of an association between values of LPS-induced cytokine not normalized for monocyte count and delirium revealed increased release of IL-8 and IL-10 in delirious patients. Increased production of IL-10, the prototypic anti-inflammatory cytokine, in delirious patients supports an idea that post-stroke immunodepression is linked to delirium. Increased release of IL-8 after blood stimulation with endotoxin is consistent with the observation of elevated serum levels of IL-8 in elderly patients with delirium after hip fracture [7].

Taking together, post-stroke delirium was associated with both immunodepression and systemic inflammation reflected by elevated circulating IL-6.

Our study has several limitations. Our results relied on only single cytokine measurement. It remains unclear if resolution of delirium is associated with any change in cytokine synthesis. In this kind of study, pre-stroke ex vivo cytokine synthesis and circulating cytokine level could not be assessed. We cannot exclude a possibility that some of our patients could have delirium only in the first hours of stroke before admission. Our findings could not be generalized on non-stroke patients with delirium and require confirmation in different groups of delirious patients.

## Conclusion

In conclusion, reduced ex vivo release of pro-inflammatory cytokines after LPS stimulation and elevated plasma IL-6 are signatures of post-stroke delirium.
